# Impact of metal filings and acoustic waves on solar still performance

**DOI:** 10.1038/s41598-026-46052-5

**Published:** 2026-04-17

**Authors:** Fifi N. M. Elwekeel, Samar Mansour, Mahmoud Abdelmagied, Wael I. A. Aly

**Affiliations:** Faculty of Technology and Education, Helwan University, Cairo, Egypt

**Keywords:** Solar still, Acoustic, Metal filings, Desalination, Engineering, Environmental sciences

## Abstract

The current study investigates the parameters for improving the performance of a water stiller. The paper looks at two influencing factors. The first parameter is the effect of various metal filings at changing concentrations. The second parameter is the impact of acoustic waves of different shapes. Copper, aluminum, iron, and stainless-steel filings are used at concentrations of 25 g/l, 50 g/l, 75 g/l, and 100 g/l. Acoustic waves are sine waves, square waves, triangular waves, and sawtooth waves that have frequencies of 290 Hz, 300 Hz, and 320 Hz. The results show that the highest water productivity from this water stiller can be achieved by using copper filings at 75 g/l filing concentration. Water production rises by 8.8% when copper filings are used instead of none. Desalinating water with copper filings costs 0.57 LE per liter. Acoustic waves reduce water productivity regardless of wave shape. Triangular waves have the lowest influence. At 300 Hz, triangular waves produce an average water productivity of 86 ml, whereas the average water productivity without waves is 90.55 ml. Copper filings with a low concentration (no more than 75 grammes per liter) can boost water distiller yields while also placing the distiller in a quiet location away from sound waves.

## Introduction

The demand for fresh water has become one of life’s most necessities, particularly in recent years. The demand for fresh drinking water has increased as the population has grown. It has become one of the most important issues confronting us right now. Especially in remote areas and countries where fresh water is scarce, it is one of the most important aspects of daily living for individuals seeking to drink, eat, farm, and manufacture^1^. As a result, numerous scientists and researchers have recently shared their findings. Which investigates possibly solutions to the problem of a lack of fresh water. That is via developing alternative techniques to desalinate salt water, which makes up around 97.5% of the total water available on earth^2^. Researchers are looking for new ways to desalinate water using solar energy, which is both clean and economical. The most well-known process is distillation. Solar distillation uses heat to evaporate saltwater, which is then condensed on a cold surface and collected to produce water suitable for drinking or irrigation^3^. So, the researchers focused on studying the performance of solar stills and improving methods.

Alwan et al.^4^ conducted experiments on a single-slope solar still to investigate the effect of water depth in the basin on heat transfer and productivity. The results showed that 1 cm of water depth produced the greatest water productivity when compared to the other two depths. At 1:00 PM, the heat transfer coefficient was 33.37 W/m^2^K, with a basin water depth of 1 cm.

Omara et al.^5^ investigated the improvement of solar still productivity utilizing a water stirring fan at water depths of 1, 2, 3, and 4 cm. Their water productivity rose by almost 17% at 3 cm and 30 rpm. Akash et al.^6^ studied the inclination angles of the solar still’s cover glass. The angles were 15°, 25°, 35°, 45°, and 55°, and the results showed that the best inclination angle was 35 degrees. Ketabchi et al.^7^ investigated the effects of inclination angle on a solar still with cooling glass cover and top and bottom reflectors. The results showed that a basin tilted at 25°, with top and bottom reflectors inclined at 10° and 45°, yielded the greatest production of 4.2 kg/m^2^. Abdullah et al.^8^ modified a single basin solar still by adding trays to the interior sides and using internal and external mirrors. The redesigned solar still generated 95% more than the traditional solar still.

In recent investigations, nanofluids and hybrid nanofluids were examined to improve heat transfer properties and demonstrated a significant improvement^9–11^.

Sahu and Tiwari^12^ compared the performance of a single-slope solar still with and without nanofluids. They tested the thermal and physical properties of zinc oxide, silicon oxide, and a combination of the two at various water depths. The highest cumulative yields were found to be 2370 mL, 2630 mL, 3070 mL, and 3480 ml without nanofluids, silicon oxide nanofluids, a blend of zinc oxide and silicon oxide nanofluids, and zinc oxide nanofluids, respectively. Marzouk et al.^13^ added various compounds to the basin water of solar stills to increase their efficiency. CuO had the largest cumulative freshwater yield (4.13 L/m^2^) and produced a 137% increase in energy efficiency compared to a conventional solar still (CSS). Dhasan et al.^14^ studied the use of metal oxide nanoparticles as a coating material in absorber plates with varying water thickness. The results showed that the fresh water created from solar stills without an absorber coating for water thicknesses of 10, 20, and 30 mm was 3.85, 3.09, and 2.90 kg/m^2^, respectively. However, with good coating of the absorber plate using metal nanoparticles, the cumulative daily water generated from the solar still for varied water thicknesses of 10, 20, and 30 mm increased to around 6.03, 5.36, and 4.72 kg/m^2^, respectively. Saad et al.^15^ combined longitudinal fins embedded in a phase change material (PCM) layer with a solar still. The study’s findings showed that embedding longitudinal highly conductive fins into PCM enclosures enhanced the contact surface area between the basin’s backside and the PCM, improving thermal energy storage and recovery rates. During the day, their system increased thermal efficiency by 45.12% and yielded clean condensed water by 51.13%. Abdullah et al.^16^ investigated the effects of coating the surfaces of a solar still with copper oxide nanoparticles as a nano coating and using reflectors and PCM. The total freshwater yield of the solar distiller was improved by 14 and 108% when CuO nanoparticles were used in paint, as well as the collection of reflectors, nano coating, and PCM with CuO nanoparticles, compared to the reference still. In addition, titanium nanoparticles in the black coating for the absorber surface increased water production; the average daily productivity of the solar still was 7.89 L throughout the summer^17,18^.

Kabeel et al.^19^ compared a conventional tubular still to a tubular solar still using copper tubes filled with phase change material. The results showed that accumulated production from conventional tubular still ranged between 4.1 and 4.31 L/m^2^/day. While using closed copper tubes filled with PCM, accumulated production increased to 8.82–9.05 L/m^2^/day, with a ratio of 110–115.1%. Younes et al.^20^ investigated the effect of nano-PCM, reflectors, and vertical wick on tubular solar collectors. Their results revealed that nano-PCM improved vertical wick productivity with reflector by 26% compared to the vertical wick with reflector. Alahmadi et al.^21^ examined three hemispherical solar stills. Incorporating planned wire meshes at three specified positions (base, middle, and water surface). The proposed wire mesh serves as both a heat storage medium and a secondary porous absorber, increasing surface area and boosting thermal energy absorption, transfer, and storage, resulting in increased overall productivity. Their findings revealed that positioning the porous absorber on the water’s surface resulted in the highest still production and optimal performance. Stills with wire mesh on water surface had a daily production of 8.06 L/m^2^. Kabeel et al.^22,23^ studied the impact of graphite plates or graphite nanoparticles with paraffin wax absorber plates in a solar still. Experimental investigation showed that employing graphite resulted in a maximum improvement in water production of 94.52%. Abu-Arabi et al.^24^ investigated hybrid solar stills. They evaluated three systems. They had three systems: solar still with cooling the glass cover, solar still with solar water collector and cooling the glass cover, and solar still with solar water collector, cooling the glass cover, and PCM materials. Productivity increased by 1.8 times for solar stills with cooling the glass cover and solar water collector, and 2.3 times for solar still with cooling the glass cover, solar water collector, and PCM when compared to solar still with cooling the glass cover system.

Wicks made of linen, cotton, or jute can also be added to the salt water to improve heat transfer, hence enhancing the still’s efficiency and production^25,26^. Biswal et al.^27^ compared a single-slope solar stiller equipped with heat storage materials to one that was not modified. Wick materials, gravel, and sand were used as storage materials to improve evaporation rates and hence increase freshwater production. Their results showed that cumulative productivity increased by 55.281, 20.722, and 39.393% for water inputs of 15, 20 and 25 L, respectively, compared to conventional solar desalination systems. Alshqirate et al.^28^ evaluated the use of a solar still equipped with palm leaves in the basin to increase evaporation rate and thus water productivity. They discovered that adding natural fibers improved water yield by 44.5% when compared to a solar still without palm leaves. Dumka et al.^29^ created a modified solar still with 100 cotton bags filled with sand and compared it to a non-modified solar still at basin water levels of 30 kg and 40 kg. The cumulative distillate production of the improved solar still for 30 and 40 kg basin water was 28.56 and 30.99% higher than the nonmodified solar still. Hammoodi et al.^30^ studied the effects of different types of wick materials as absorbers on water productivity from solar stills. The wick materials used included light cotton cloth, heavy cotton cloth, jute cloth, and velvet cloth. When they used a light cotton wick in a solar still, productivity increased by 11%, 24.2%, 76.7%, and 136.6% compared to solar stills integrated with heavy cotton, jute fabric, velvet fabric, and traditional solar still, respectively. Nijmeh et al.^31^ utilized materials to increase the absorptivity of water to solar radiation. The materials used were dissolved salts (potassium permanganate and potassium dichromate), violet dye, and charcoal. The addition of potassium permanganate led to a 26% increase in efficiency.

Solar still distillation using waste thermal energy was employed to boost evaporation and water output. The greatest water production and thermal efficiency of solar still with wasted energy were 17.13 L/m^2^ and 34.2%, respectively^32^. Sampathkumar and Senthilkumar^33^ studied the effects of water heating before feeding it to the still. They used a solar still and an evacuated tube collector to heat the water. When the system was combined with a 24 h period, the water productivity increased by double. Patel et al.^34^ studied the effects of integrating an external partial cooling coil condenser with a solar still during summer and winter sessions. In the summer, the greatest daily yield was 11,499 ml/day, while in the winter, it was 8212 ml/day, with a daily system efficiency of 76.66% and 54.74%, respectively. Shukla et al.^35^ created an evacuated tube collector integrated serpentine-based active desalination system to provide potable water and hot water. They assessed their system based on energy, exergy, and economic analyses. At a water depth of 0.03 m, measurements found maximum daily water productivity, energy efficiency, and exergy efficiency of 5.10 kg/m^2^ day, 42.39%, and 4.45%, respectively.

This acoustic approach was utilized to reduce turbidity and suspended particles, but not for water desalination. Chan et al.^36^ employed low and high MHz to treat greywater samples. They improved the treatment procedure by 24% in just two hours. In 5 s, sonic water treatment reduced turbidity by 5–7 times^37^.

Metals were used as nanomaterials in previous studies. Metals increase heat transfer and the performance of water still. There has been no research comparing the effects of metal filings on solar stills. Even though these filings are waste products from operational workshops. As a result, this work presents solar still desalination with copper, aluminum, iron, and stainless-steel filings, as well as an investigation into the best concentration of each filing. Sound waves have been utilized to promote heat transfer in heat exchangers^38^, but not in the use of water distillation equipment. As a result, the current study included investigation into how sound waves affected the performance of water distillation apparatus. The present work depicts four acoustic wave shapes with varying frequencies.

## Experimental setup and procedures

**Fig. 1:**
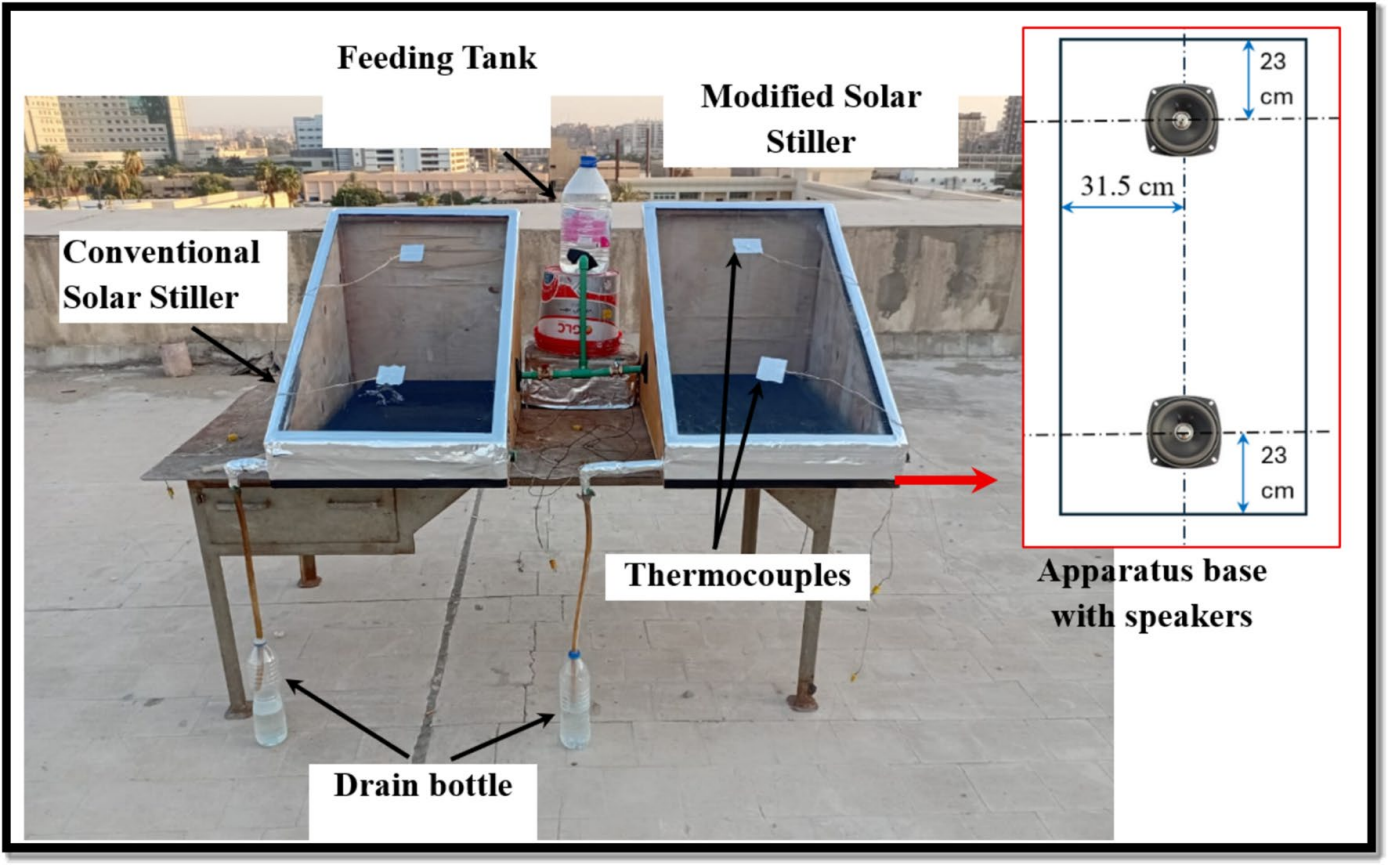
Photograph of the experimental setup.

Figure 1 depicts an image of the experimental solar still. The apparatus is traditional single-slope solar still. It is built of 1.8 cm thick plywood, 86.6 cm long, 60 cm wide, and 60 cm high, with a 0.5 cm thick glass cover with a 30° slope. The basin is 69 cm long, 48 cm wide, and 5 cm high. It is made of rust-resistant galvanized sheet metal and coated with black varnish to absorb maximum sunlight as possible. The concept of solar stills depends on the sun’s rays falling on the glass surface and passing through it into the basin. The salt water in the basin heats and evaporates, and the vapor rises to the glass cover in preparation for condensation. The condensed water slides in the direction of the inclined glass surface and is collected through a collection channel. The experiments consist of 242 runs. The experiments were conducted from 8:00 a.m. to 18:00 p.m. in July 2024 at Helwan University’s Faculty of Technology and Education (30.10N, 31.29E), Cairo.

**Fig. 2:**
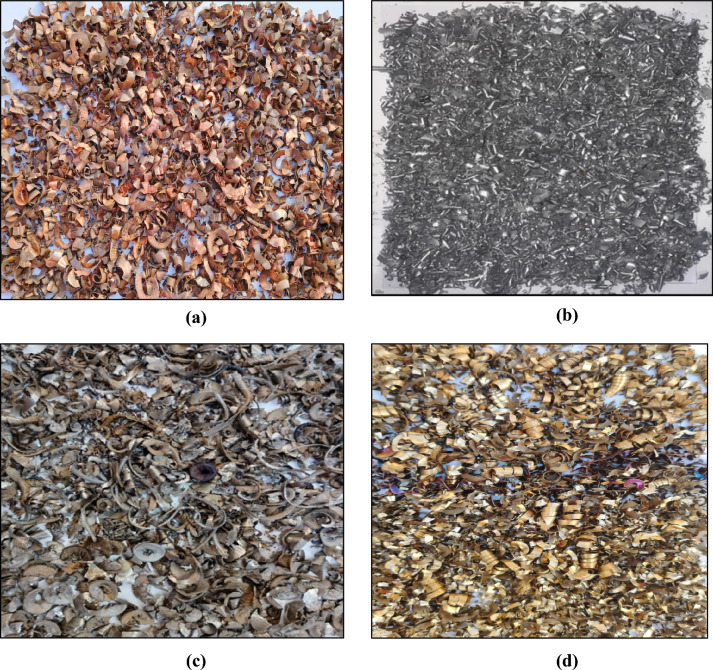
Different types of metal filings (**a**) Copper filings, (**b**) Aluminum filings, (**c**) Iron filings, and (**d**) Stainless steel filings (AISI 316).

The experimental techniques include two ways for investigating the productivity of a solar still. The first method employs various metal filings (see Fig. 2) such as copper, aluminum, iron, and stainless steel at concentrations in saltwater of 25 g/l, 50 g/l, 75 g/l, and 100 g/l. The second method involves employing sound with frequencies of 290, 300, and 320 Hz, as well as various wave types such as sine, square, triangle, and sawtooth. Sound is transferred to seawater by installing two speakers on the basin, each having 13 cm in diameter and 0.9 W. Five thermocouples are installed to measure temperature, and they are distributed as follows: two on the glass surface to measure surface temperature, and three in the basin to measure basin temperature.

## Uncertainty analysis

Solar radiation, temperature, wind speed, water productivity, and metal filing weight are all measured in the experiment. According to the parameters of each measuring device as indicated in Table 1, the standard uncertainty is computed using the following equation^39^:

1 $$u=\frac{a}{\sqrt{3}}$$

where a and u are accuracy and uncertainty, respectively.

**Table 1:** Range, accuracy, and uncertainty of measured parameters.

Parameter	Devices	Range	Accuracy	Standard uncertainty
Thermocouples	K-type thermometer	−50:1300 °C	$\pm$ 0.1	0.0577
Solar radiation	Digital solar power meter (TM-206)	0:2000 W/m^2^	$\pm$ 0.1	0.0577
Air velocity	Digital anemometer (GE-2409015)	0:30 m/s	$\pm$ 0.15	0.0866
Productivity	graduated bottle	0–1500 mL	$\pm$ 1.5	0.8660
Weighing metal filings	Digital scale (SF-400)	0:10000gm	$\pm 1$	0.5773

## Methodology

### Thermal analysis

The hourly thermal efficiency of a solar still is a ratio of the energy required to desalinate water per hour to the input rate of solar energy per hour, which is determined using the accumulated amount of distilled water over the day and the total incident solar energy throughout the day. The thermal efficiency is computed as follows^40^:

2 $${\eta }_{d}=\frac{\sum {m}_{d}\times {h}_{fg}}{\sum {A}_{abs}\times I\left(t\right)\times 3600}$$

where h_fg_ is latent heat, which is calculated using the average water temperature (${\mathrm{T}}_{\mathrm{w},\mathrm{av}})$ inside the basin and represented by^41^.

3 $$h_{fg} = {3}.{1625} \times 10^{6} + \left[ {1 - \left( {7.616 \times 10^{ - 4} \times T_{w,av} } \right)} \right]\;{\mathrm{for}}\;\user2{ T}_{{{\boldsymbol{w}},{\boldsymbol{av}}}} > 70$$

4 $$h_{fg} = 2.4935 \times 10^{6} \left[ \begin{gathered} 1 - \left( {9.4779 \times 10^{ - 4} \times T_{w,av} } \right) + \left( {1.3132 \times 10^{ - 7} \times T^{3}_{w,av} } \right) \hfill \\ - \left( {4.7974 \times 10^{ - 9} \times T^{2}_{w,av} } \right) \hfill \\ \end{gathered} \right]\;{\mathrm{for}}\user2{ T}_{{{\boldsymbol{w}},{\boldsymbol{av}}}} < 70$$

### Cost analysis

The cost per liter of desalinated water (CPL) can be computed using the following Eq. (5)^42,43^:

5 $$CPL=\frac{{T}_{C}}{TFP}$$

where T_C_ and TFP represent the total cost and total desalinated water produced during the life of the water stiller, respectively.

The total cost can be defined as a function of fixed cost (F_c_) and variable cost (V_c_), as follows:

6 $${T}_{c}={F}_{c}+{n\times V}_{c}$$

where n is the water stiller’s estimated life of ten years. The variable cost is 30% of the fixed cost^42^. It can be calculated as follows:

7 $${V}_{C}=0.3\times {F}_{c}$$

The total desalinated water generated during the life of the water stiller can be computed as follows:

8 $$TFP=m\times Operating days$$

where m is the average daily production and operational days are the number of days the water stiller operates over a ten-year period, as one year in Egypt is around 340 days^45^.

## Results and discussion

### Weather conditions

**Fig. 3:**
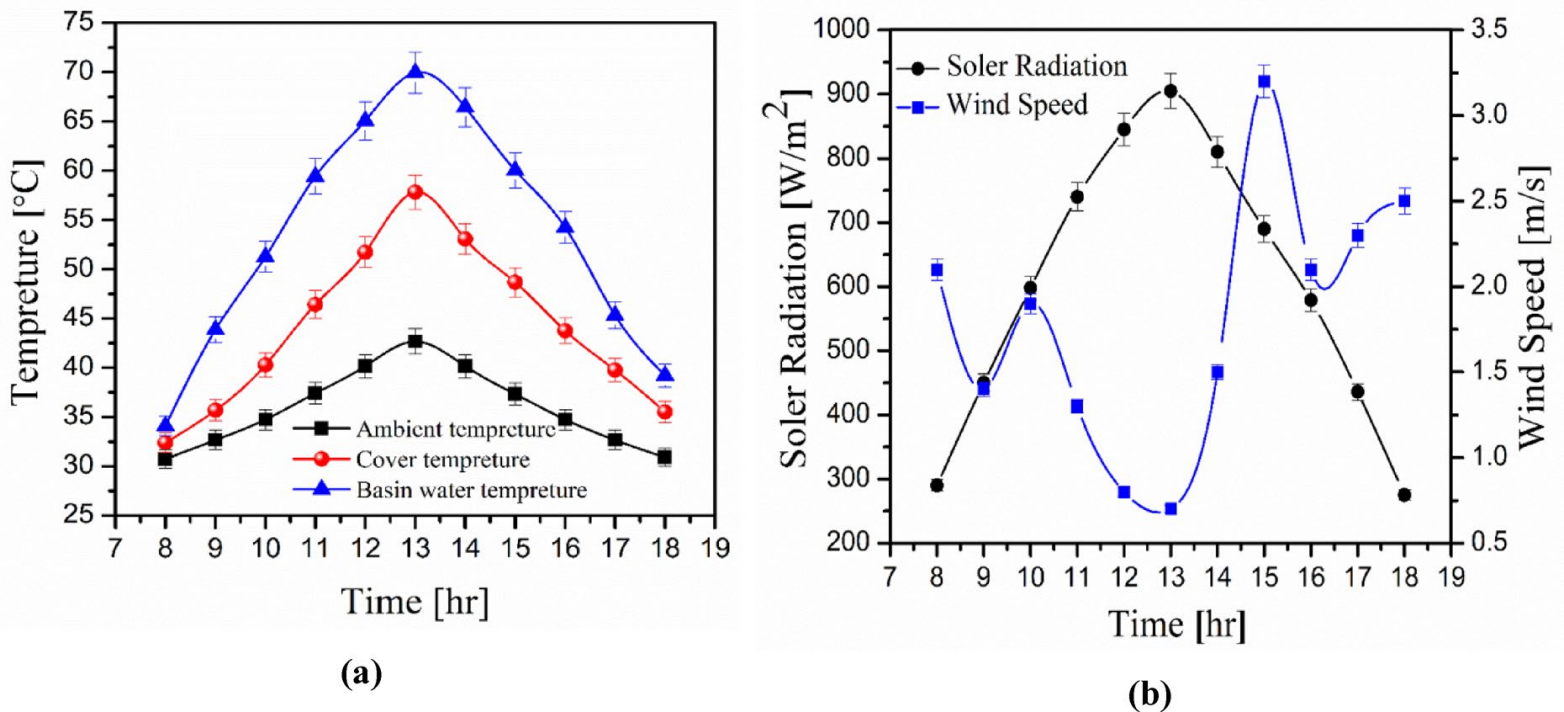
Climatic condition: (**a**) Solar radiation and wind speed, (**b**) basin Water temperature, Surface temperature, Ambient temperature.

Figure 3 depicts the measurement of solar radiation during the experiment days. It starts at 300 W/m^2^ at 8:00 a.m. and rises to its peak at 1:00 p.m., when it reaches 890 W/m^2^, before gradually decreasing to 260 W/m^2^ at the end of the experiment at 18:00 p.m. On the other hand, the wind speed is represented in this figure, and it changes during the day. The wind speed varies from 0.3 to 4 m/s.

**Fig. 4:**
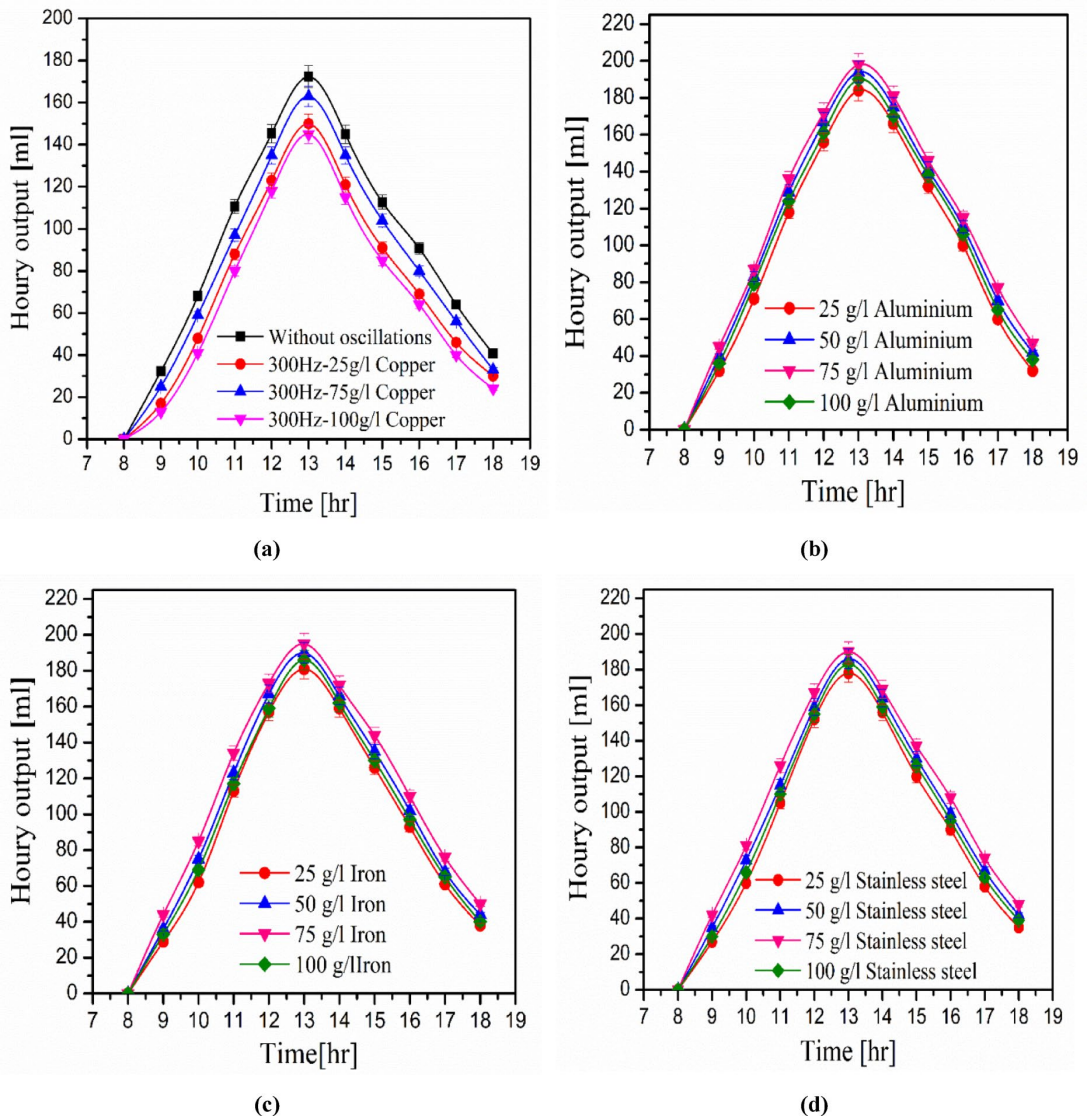
Water productivity with and without metal filings concentrations, (**a**) Copper filings, (**b**) Aluminum filings, (**c**) Iron filings, and (**d**) Stainless steel filings.

Figure 4 shows the ambient temperature, glass cover temperature, and basin water temperature across the experiment hours. Temperatures rise slightly from 8 a.m. to the highest value at 1:00 p.m., then fall to low values. The basin water temperature and ambient temperature provide the maximum and lowest temperatures, respectively. At 1:00 pm, the maximum values for ambient temperature, glass cover temperature, and basin water temperature are 48 °C, 64 °C, and 73 °C, respectively.

### Effects of types of metal filings

**Fig. 5:**
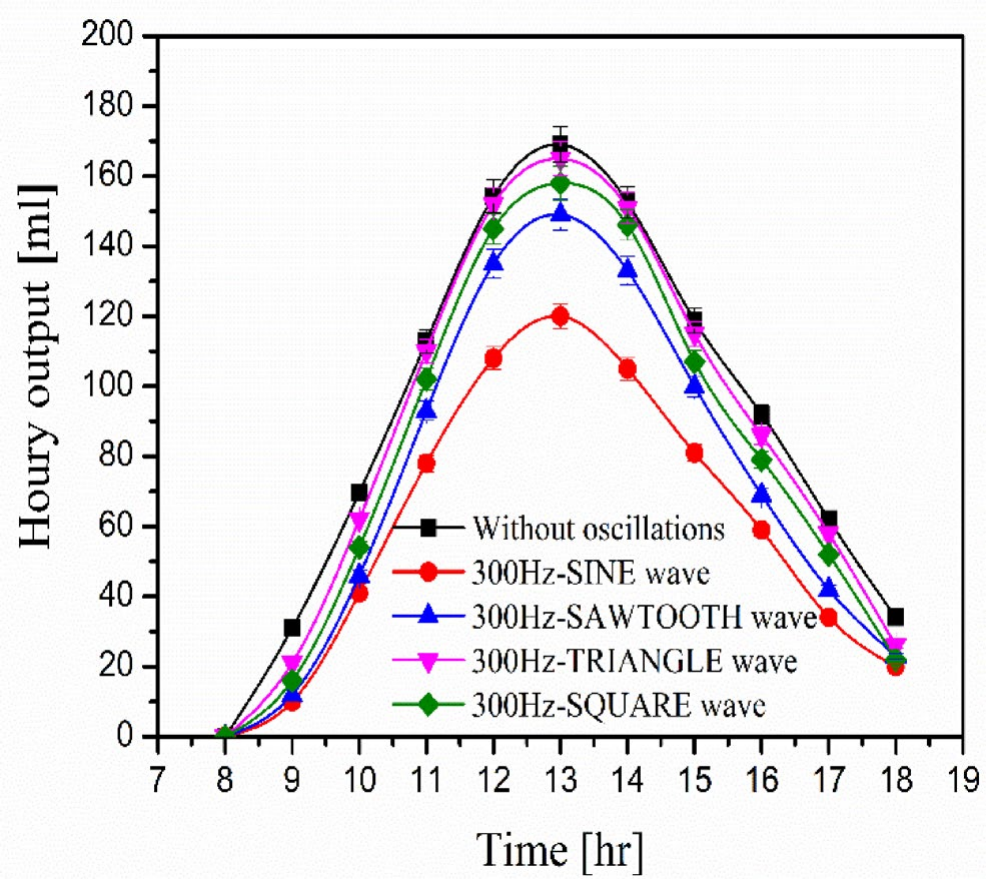
Relation between water productivity and acoustic waves.

Figure 5 displays the hourly output of desalinated water at various metal filing concentrations. Starting in the morning, the production of water grows every hour until it reaches its peak at midday, at which point it steadily falls until the experiment ends at 18:00. This reflects both the behavior of solar radiation and the temperature of the water in the basin. Water productivity with metal filings is significantly higher than without them. This is because metal filings raise water temperatures, which increases evaporation and condensation rates. This fig. also shows that all concentrations of metal filings enhance water productivity except for the 100 g/l concentration, which may be because the high concentration of metal filings impedes heat transfer between water layers, resulting in a lower condensation rate.

Table 2 shows average productivity during the day and various study metal filings concentrations. Copper has great productivity over a range of metal filing concentrations, as demonstrated by its high heat conductivity. The highest and minimum average productivities for copper and stainless steel are 114.27 ml and 93.27 ml, respectively, at a metal filing concentration of 75 g/l. When a copper filing concentration of 75 g/l is used, water productivity increases by 8.8% compared to the case without copper filings.

**Table 2:** Thermal conductivity^44^ and average water productivity for metal filings [ml].

Items	Concentrations [g/l]				K [W/m. K] @T = 27 °C
	25	50	75	100	
Copper	97	106.27	114.27	101.73	401
Aluminum	95.55	104.64	109.45	100.64	237
Iron	92.64	100.55	107.55	96.18	80.2
Stainless steel (AISI316)	89.18	97.27	103.82	93.27	13.5
Without metal filings	88.90				–

### Effects of acoustic waves on water productivity

**Fig. 6:**
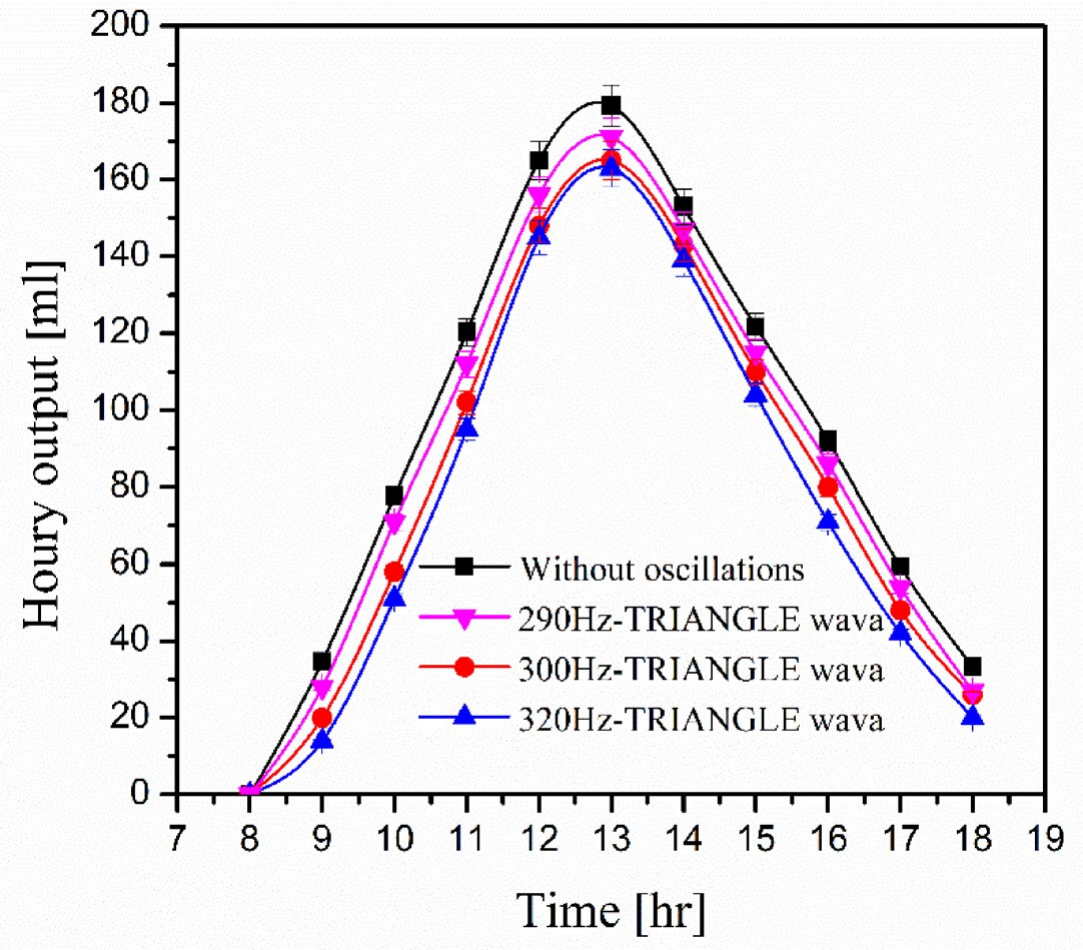
Relation between water productivity and various values of triangular waves.

This study investigates how the four types of oscillatory waves affect water productivity. The four acoustic waves are sine, sawtooth, triangle, and square waves. They have a constant frequency of 300 Hz. Figure 6 depicts water productivity during the day hours with and without oscillatory waves. The water productivity increases during the day and reaches its maximum at noon 13:00. The shape of the oscillating waves affects water productivity, and oscillation reduces water productivity for all oscillating wave shapes. This can be explained by the oscillating waves hindering heat waves, which influence the quantity of heat absorbed by the basin wall. As a result, the temperature of the water inside the basin drops, lowering the rate of condensation. Triangular waves, square waves, saw waves, and sine waves have average water productivities of 86 ml, 80.09 ml, 72.91 ml, and 59.64 ml at 300 Hz, respectively. While the average water productivity without oscillatory waves is 90.55 ml. The triangle wave has the least effect on water production, as it is near to the water productivity curve without oscillations.

**Fig. 7:**
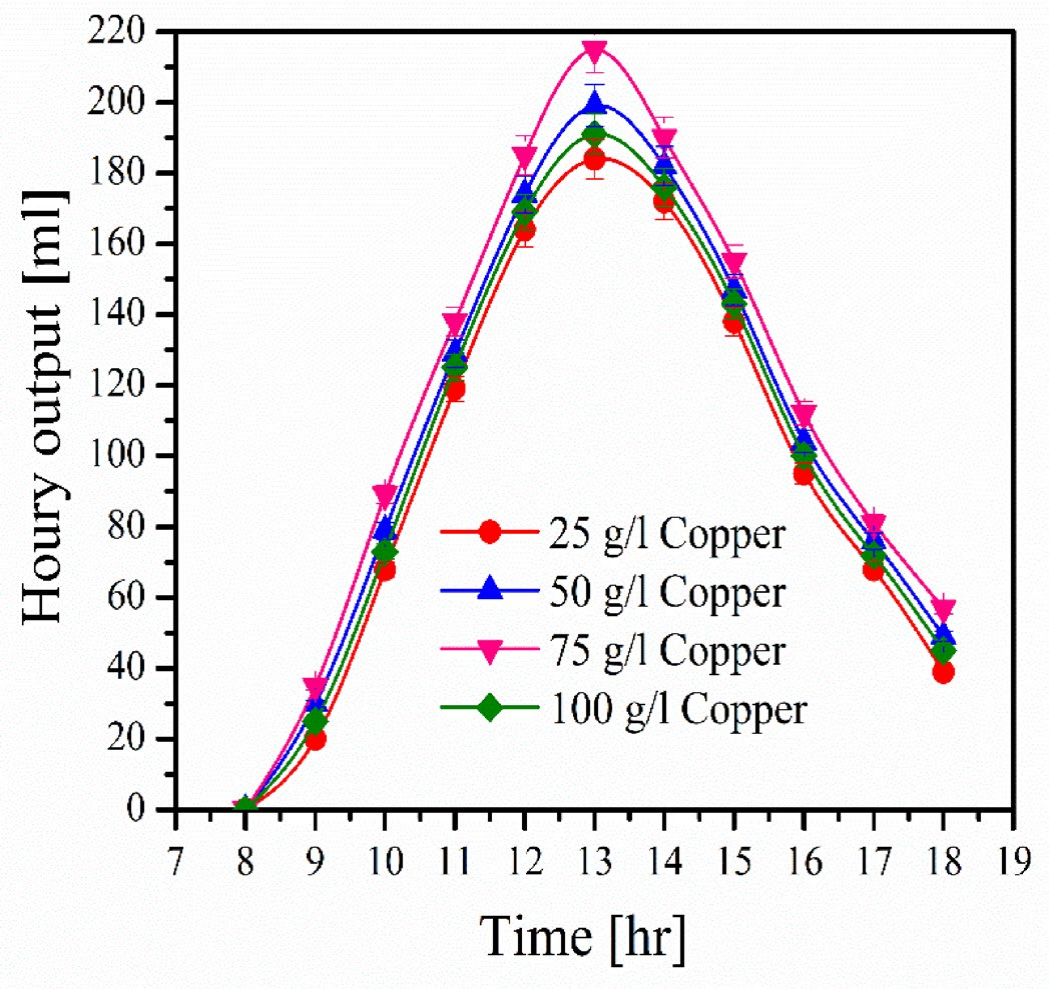
Relation among water productivity, copper filings concentration, and oscillations.

Figure 7 depicts the influence of triangle wave frequencies on water productivity. It shows three frequency values: 209 Hz, 300 Hz, and 320 Hz. Water production decreases as frequency rises. High frequencies impede solar heat waves because high frequency of acoustic waves travel in all directions^47^. It raises the impedance of solar heat waves, which reduces the condensation rate and, so, water production. The average water productivity during the day is reduced by 6.7%, 13.2%, and 18.6% at frequencies of 290 Hz, 300 Hz, and 320 Hz, respectively, as compared to water productivity without oscillation.

**Fig. 8:**
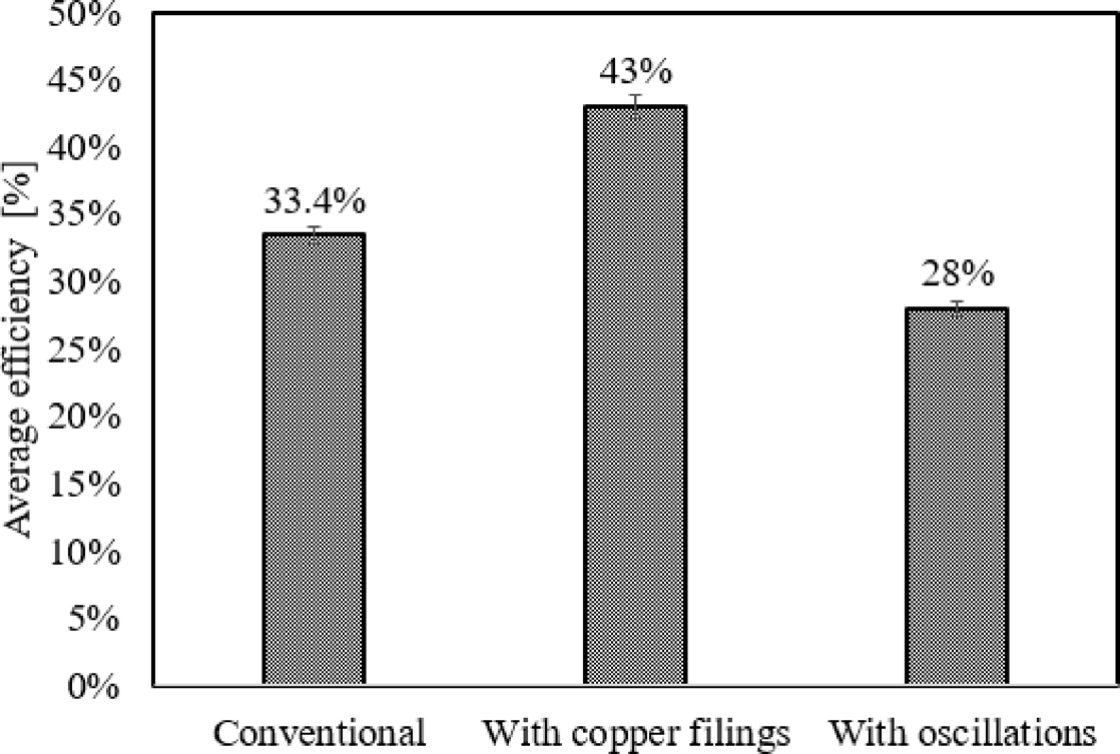
Solar stiller efficiency with and without improvements.

Figure 8 demonstrates the effect of copper filings concentrations using a triangle acoustic wave on water productivity in a distiller. The acoustic frequency eliminates the enhancement from copper filings. This figure also shows that with a high concentration of copper filings, the distiller produces the least amount of water in the presence of acoustic frequency. The average water productivity without oscillations is 89.24 ml, while with copper filings of 25 g/l, 75 g/l, and 100 g/l at a frequency of 300 Hz, the average water productivity is 71.18 ml, 80.64 ml, and 65.91 ml, respectively.

## Thermal efficiency and cost

**Fig. 9:**
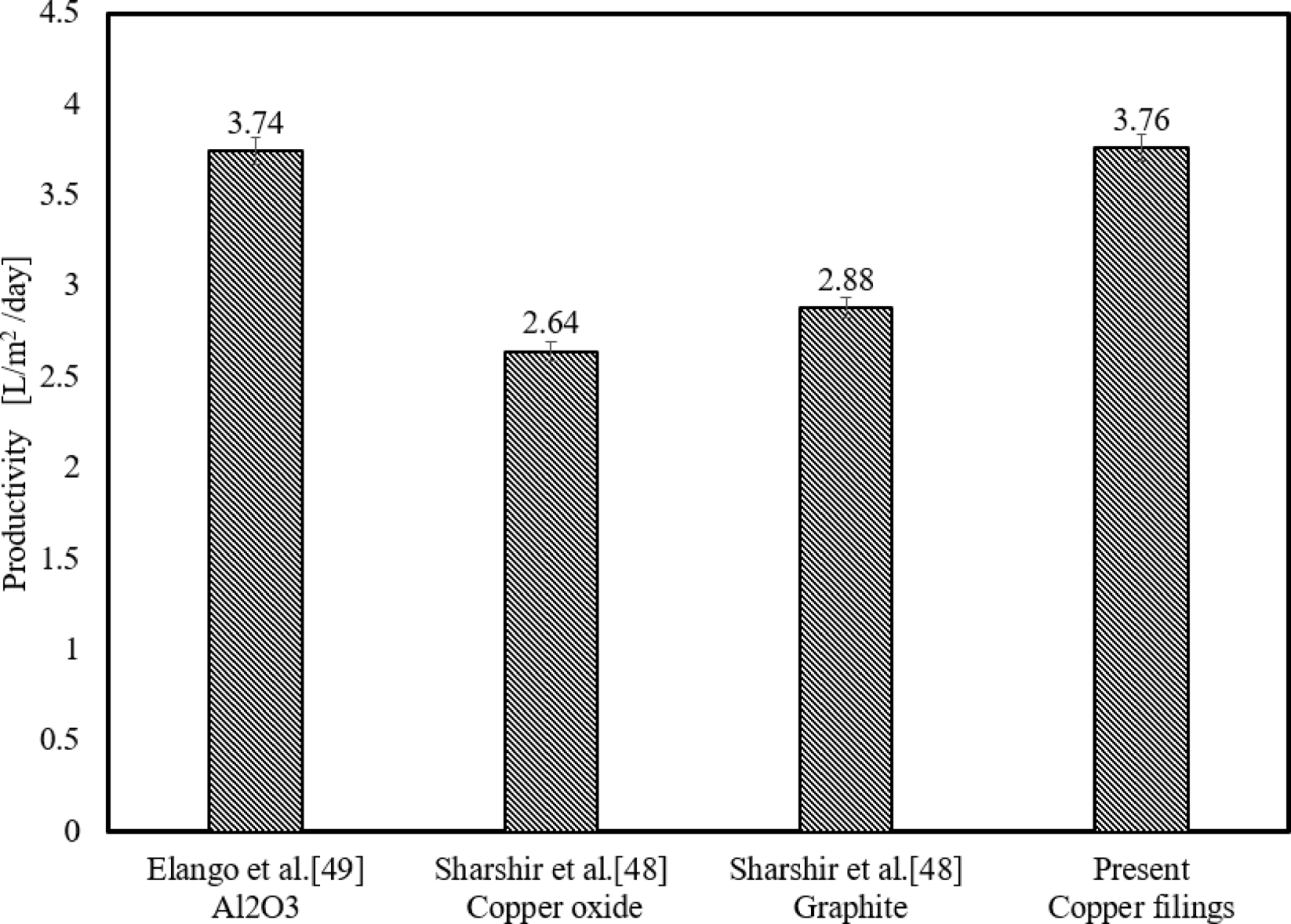
Water Productivity Comparison.

Figure 9 compares the average thermal efficiency of a solar distiller with and without copper filings and acoustic waves. The average thermal efficiency of a solar distiller with copper filings is calculated for all runs using a copper filing concentration of 75 g/l. The average thermal efficiency of a solar distiller for four acoustic waves is computed for all runs at 300 Hz. The solar distiller with copper filings has the highest performance, reaching 43%. Acoustic waves have the lowest average thermal efficiency of a solar distiller (28%).

Table 3 compares the costs of a liter of desalination water with and without copper filings. The cost analysis is carried out at a copper filing concentration of 75 g/l. The table demonstrates that the cost of desalinating water per liter using copper filings is 0.57 Egyptian pounds per liter, although the total cost using copper filings is higher than without them. That is due to the high productivity of water when using copper filings, which outweighs the total cost.

**Table 3:** Cost of destination with and without copper filings.

Items	Conventional	With copper filings
Total cost, ${T}_{c}$	5700 LE	7250 LE
TFP	9792 L	12,784 L
CPL	0.58 LE/L	0.57 LE/L

## Comparison of the current study to previous research

**Fig. 10:**
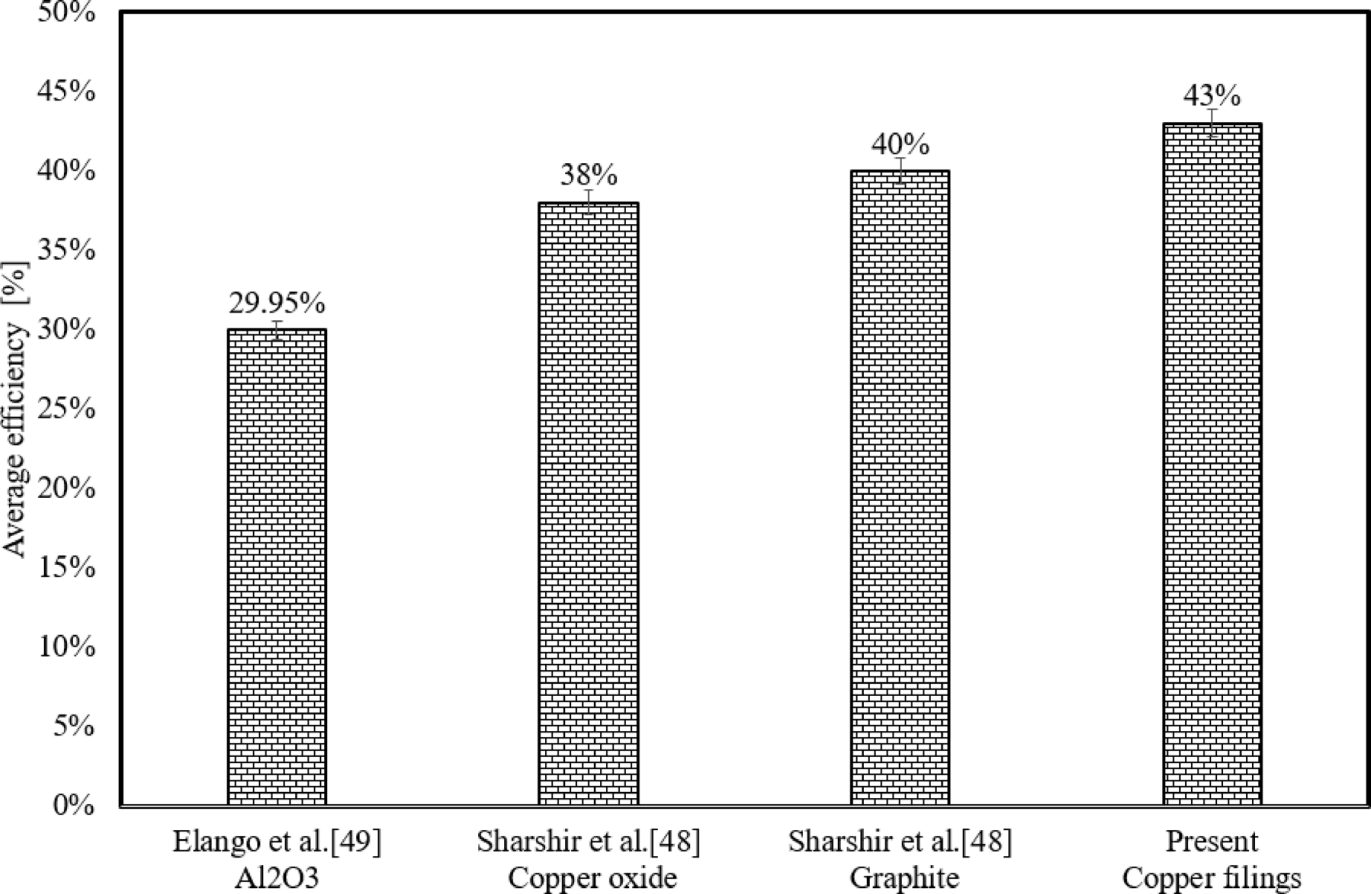
Average Thermal Efficiency Comparison.

Figure 10 and show comparisons of water productivity and average thermal efficiency between this study and previous investigations. Previous studies used nanomaterials such as alumina, copper oxide, and graphite. The average water productivity in the present study utilizing copper filing is the highest, reaching 3.76 L/m^2^/day. Furthermore, the average thermal efficiency of the system with a copper filing concentration of 75 g/l is higher than that of previous research, reaching 43%. This ratio can reach high levels when scrap metal is purchased at a low cost.

## Conclusions

This study examines the factors that affect the performance of a solar still. The first effect is the addition of metal filings at various concentrations, whereas the second effect is caused by acoustic waves. Copper, aluminum, iron, and stainless steel are used as metal filings. The metal filings concentrations are 25, 50, 75, and 100 g/l. Acoustic waves include sine waves, square waves, triangular waves, and sawtooth waves with frequencies of 290 Hz, 300 Hz, and 320 Hz. Experiments are carried out in the climate of Cairo, Egypt. The experimental results can be summarized as follows.

- In comparison to the other metal filings examined, copper has the highest productivity at metal filing concentrations.
- Copper and stainless steel have maximum and minimum average productivities of 114.27 ml and 93.27 ml, respectively, at a metal filing concentration of 75 g/l.
- When a 75 g/l concentration of copper filings is employed, water productivity increases by 8.8% when compared to without the use of copper filings.
- Acoustic waves decrease water productivity regardless of wave shape. At 300 Hz, triangular waves, square waves, saw waves, and sine waves have average water productivities of 86 ml, 80.09 ml, 72.91 ml, and 59.64 ml, respectively, while the average water productivity without waves is 90.55 ml.
- Water production drops as frequency increases. Water production during the day is lowered by 18.6% at a frequency of 320 Hz when compared to water productivity without oscillation.
- Copper filings provide the highest average thermal efficiency of a solar distiller, reaching 43%, while acoustic waves provide the lowest average thermal efficiency (28%).
- The cost of desalinating water per liter with copper filings is 0.57 Egyptian pounds per liter, while desalinating water without copper filings is 0.58 Egyptian pounds per liter.

Finally, low-concentration copper filings (no more than 75 grammes per liter) can improve water distiller yields. Water distillers work poorly when exposed to sound waves, therefore place the distiller in a quiet location.

## Data Availability

The data supporting the findings of this study can be obtained from the corresponding author upon request, subject to reasonable conditions.

## List of symbols

$A_{abs}$: Total absorption area, m^2^
$$a$$: Accuracy
$CPL$: Cost per liter, LE
$F_{C}$: Fixed cost, LE
$h_{fg}$: Water latent heat of vaporization, J/kg
$I\left( t \right)$: Total solar irradiance, W/m^2^
$m_{d}$: Total fresh water produced per hour, kg/h
$n$: Still lifetime, years
$T_{C}$: Total cost, LE
T_w_: Temperature of saltwater in the basin, ^o^C
$TFP$: Total fresh water produced, L
t: Tim, hr
$$u$$: Uncertainty
$V_{C}$: Variable cost, LE

## Greek letters

$${\eta }_{d}$$: Thermal efficiency,

## Abbreviations

PCM: Phase change materials
